# Evaluating the Effects of Combined Transcutaneous Spinal Direct Current Stimulation and Exercise Therapy on Pain and Psychological Symptoms in Fibromyalgia Patients: A Prospective Randomized Study

**DOI:** 10.3390/biomedicines14092007

**Published:** 2026-09-07

**Authors:** Angelica De Sandi, Daniele Saccenti, Maurizio Vergari, Denise Mellace, Eleonora Zirone, Giada Aglieco, Martina Nigro, Giorgia Boboni, Stefano Zago, Filippo Cogiamanian, Gabriella Pravettoni, Alberto Priori, Roberta Ferrucci

**Affiliations:** 1 Fondazione IRCCS Ca’ Granda Ospedale Maggiore Policlinico, 20122 Milan, Italy; angelica.de.sandi@gmail.com (A.D.S.); maurizio.vergari@policlinico.mi.it (M.V.); eleonora.zirone@policlinico.mi.it (E.Z.); giada.aglieco@policlinico.mi.it (G.A.); martina.nigro@policlinico.mi.it (M.N.); giorgia.boboni@studenti.unimi.it (G.B.); stefano.zago@policlinico.mi.it (S.Z.); filippo.cogiamanian@policlinico.mi.it (F.C.); 2 Department of Oncology and Hemato-Oncology, University of Milan, 20122 Milan, Italy; daniele.saccenti@unimi.it (D.S.); denise.mellace@unimi.it (D.M.); gabriella.pravettoni@ieo.it (G.P.); 3 Division of Applied Research for Cognitive and Psychological Sciences, IEO European Institute of Oncology, 20141 Milan, Italy; 4 Department of Health Science, University of Milan, 20146 Milan, Italy; alberto.priori@unimi.it; 5 Azienda Socio Sanitaria Territoriale Santi Paolo e Carlo, 20142 Milan, Italy

**Keywords:** fibromyalgia, transcutaneous spinal direct-current stimulation, exercise therapy, pain, anxiety, depression, quality of life

## Abstract

**Background**: Chronic pain syndromes such as fibromyalgia have been associated with worsening health-related quality of life and increased incidence of comorbid psychiatric disorders, underscoring the need for targeted interventions. Transcutaneous spinal direct-current stimulation (tsDCS) and exercise therapy (ET) have been identified as promising non-pharmacological tools due to their capacity to inhibit pain transmission. However, empirical evidence regarding a combined efficacy remains limited. The aim of this work was, therefore, to test the effects of a multimodal treatment protocol on pain and psychological symptoms. **Methods**: Forty-five patients with fibromyalgia were recruited and randomly assigned to the tsDCS, ET, or combined tsDCS + ET group for a three-week period. Participants were assessed at baseline, one week after the start of treatment, at the end of the treatment (T2), and at 3-month follow-up (T3) using the Visual Analogue Scale (VAS), Brief Pain Inventory (BPI), Short-Form Health Survey (SF-12), and Hospital Anxiety and Depression Scale (HADS). **Results**: Analysis of variance applied to linear mixed-effect models showed significant group-by-time interaction effects on both HADS domains (anxiety: *p* = 0.004; depression: *p* = 0.028). Post hoc pairwise comparisons highlighted that tsDCS + ET decreased subjects’ anxiety at both T2 (*p* = 0.002) and T3 (*p* < 0.001) compared to baseline. Yet, significant comparisons were not found for depression. No significant interactions were detected on VAS, BPI, and SF-12 scores. **Conclusions**: Although between-group differences were not observed, the integration of neuromodulation with physical activity resulted in a significant improvement in patients’ anxiety-related symptoms severity, suggesting a potential added benefit.

## 1. Introduction

Fibromyalgia (FM) is a chronic pain condition characterized by widespread musculoskeletal pain, fatigue, allodynia, hyperalgesia, and sleep disturbances [1]. Its global prevalence ranges between 2% and 4% with a marked predominance in women (female-to-male ratio ~ 7:1) [2]. FM significantly impairs health-related quality of life, affecting activities of daily living, work performance, parenting, interpersonal relationships, and mental health [3,4]. Accordingly, younger individuals with FM often report a poorer quality of life compared to peers with other chronic pain conditions, with higher rates of dysfunctional behaviours, disability, school absenteeism, and healthcare services utilization [5]. Psychiatric comorbidities are also highly prevalent in FM patients, including depression, anxiety, and social isolation [6]. Notably, at least 30% of people with chronic pain experience major depression, and a similar proportion present with anxiety disorders [7]. Moreover, individuals with FM show increased suicidal ideation and a higher risk of suicide compared to healthy controls [8].

Although disruptions in nociceptive transmission are considered key mechanisms in FM [9], its pathophysiology remains poorly understood and the diagnosis relies exclusively on clinical criteria due to the lack of reliable biomarkers or instrumental diagnostic tools [10]. Hence, clinical scales play a pivotal role in quantifying pain severity, its impact on daily functioning, and associated psychological symptoms. Current treatments for FM are primarily pharmacological and largely symptomatic (e.g., tricyclic antidepressants, serotonin and noradrenaline reuptake inhibitors, anticonvulsants, myorelaxants, and analgesics) [11], with potential adverse effects and no curative options available [1]. These shortcomings highlight the need for effective non-pharmacological interventions targeting the potential underlying mechanisms of the disorder.

In this regard, non-invasive neuromodulation techniques represent a cutting-edge approach to pain management due to their ability to induce analgesia while reversing maladaptive neuroplastic changes [12]. Accordingly, these interventions have shown promising effects on both chronic pain and associated psychological symptoms [13,14]. In this framework, transcutaneous spinal direct-current stimulation (tsDCS) has gained increasing attention due to its capacity of modulating both spinal and supra-spinal excitability in a polarity-dependent manner [15]. Notably, when applied over the thoracic or cervical spinal cord, tsDCS has been shown to influence somatosensory [16], motor [17,18], and nociceptive processes [19,20], as demonstrated by the depression of the lower limb flexion reflex and modifications in corticospinal pathway activation [19,21,22,23]. TsDCS can also alter trans-synaptic efficacy [18,24], thus perturbating spontaneous neuronal activity within the lemniscal and spinothalamic pathways via plasticity-related glutamatergic processes [23,25,26]. Although its mechanisms of action are still to be fully elucidated, such consistent evidence highlights that tsDCS can modulate central nociceptive transmission at the spinal level, thereby affecting pain perception in chronic pain conditions [27,28,29].

Exercise therapy (ET), particularly combining aerobic and resistance training [30], has also been shown to ameliorate pain perception and quality of life in FM individuals [31,32]. It has been hypothesized that these therapeutic effects could be mediated by endogenous analgesic mechanisms [33], including the activation opioid-related pain-regulation systems and the modulation of corticothalamic excitability [34,35]. Besides activating the endogenous opioid system [36], physical exercise triggers dopamine release [37], which also regulates pain perception [38,39]. Collectively, these mechanisms provide a neurobiological rationale for the clinical application of ET as a non-pharmacological intervention aimed at alleviating pain and improving functional outcomes in patients with FM.

In light of these considerations, a multimodal approach combining neuromodulation with ET has been advanced to produce synergistic effects in chronic pain conditions. Integrated protocols coupling non-invasive brain stimulation and physical exercise have currently been tested in both neurological and psychiatric conditions [40,41,42,43], yielding positive effects on both cognitive functioning and clinical symptom severity [44]. A significant and homogeneous reduction in chronic pain favouring the combined intervention over ET alone, particularly with anodal stimulation of the motor cortex, has also been reported [45]. However, this evidence concerns the usage of brain stimulation coupled with physical activity, thus leaving the combined effects of spinal stimulation and ET in individuals with FM unexplored. The present study aims, therefore, to compare the effects of tsDCS, ET, and their combination on pain perception and psychological symptoms in FM subjects.

## 2. Materials and Methods

### 2.1. Participants

This prospective randomized study was conducted at IRCCS Ca’ Granda Ospedale Maggiore Policlinico (Milan, Italy). A total of 62 patients with FM were initially enrolled in the study. Seventeen participants were excluded based on predefined exclusion criteria, resulting in a final sample of 45 patients (42 females, 3 males; mean age = 51.13 ± 12.11 years). Inclusion criteria were as follow: age ≥ 18 years; both sexes assigned at birth; a diagnosis of FM according to established clinical criteria [46,47]; a score > 4 (out of 10) on the Visual Analogue Scale for perceived pain; sufficient global cognitive functioning (i.e., Montreal Cognitive Assessment score ≥ 15.50) [48] and motor abilities to participate in a rehabilitation program. Conversely, exclusion criteria were the following: changes in pharmacological therapy during the study period or initiation of new treatments within the previous month; presence of implanted electronic medical devices (e.g., brain stimulators); cranial bone defects; untreated recurrent seizures; respiratory, cardiac, or metabolic conditions incompatible with low-to-moderate-intensity physical exercise; aphasia, dementia, or severe cognitive decline; psychiatric comorbidities that interfere with compliance with a rehabilitation program; blindness or acute ophthalmological conditions that prevent the use of electronic devices with a display; and participation in other clinical trials involving pharmacological or rehabilitative interventions. The study was conducted in accordance with the Declaration of Helsinki and was approved by the ethics committee of Fondazione IRCCS Ca’ Granda Ospedale Maggiore Policlinico (Ethics Committee Milan Area 2, protocol code: 1161_2022bis). All participants provided written informed consent prior to enrolment.

### 2.2. Experimental Protocol

Eligible patients who signed the informed consent form were assigned an alphanumeric identification code and registered in a digital rehabilitation platform (Kari, Euleria, Trento, Italy). Randomization was performed as an integral part of the patient registration process using a 1:1:1 allocation ratio. Specifically, each participant was randomly assigned to one of the following three treatment groups using the “RAND” function in Microsoft Excel (Version 2302): tsDCS group (*n* = 15), ET group (ET; *n* = 15), and combined therapy group (tsDCS + ET; *n* = 15). Group allocation was performed only after participant enrollment, ensuring that the treatment assignment was concealed at the time of enrollment.

Participants in the tsDCS group and ET group received their respective treatments three times per week. In the tsDCS + ET group, participants underwent tsDCS three times per week and ET twice per week. Such a schedule was designed to maintain the planned frequency of tsDCS while limiting the overall treatment burden associated with the combined intervention and ensuring the feasibility and tolerability of the rehabilitation program. All interventions were administered over a 3-week period. All participants were followed up for 3 months from the start of the study. Clinical outcomes were assessed using validated self-report scales at four time points: before starting the treatment (T0), 1 week after the start of treatment (T1), at the end of the 3-week intervention period (T2), and 3 months after treatment initiation (T3).

### 2.3. Transcutaneous Spinal Direct-Current Stimulation

Participants were positioned comfortably in a supine position during stimulation. tsDCS was delivered using a programmable stimulator (HDCstim™, Newronika, Milan, Italy) connected to a pair of rectangular saline-soaked sponge electrodes (10 × 8 cm and 7 × 5 cm). The anode was placed over the thoracic spinal region (T10–T12), while the cathode was positioned over the primary somatosensory cortex (i.e., 2 cm rear of Cz, according to 10–20 EEG system), in line with previously described tsDCS protocols targeting spinal nociceptive processing [29]. Stimulation was delivered at an intensity of 2 mA for 20 min per session. The stimulation protocol was well tolerated, with minimal and transient side effects reported.

### 2.4. Exercise Therapy

ET was performed at home using the digital rehabilitation system (Kari, Euleria, Trento, Italy) for approximately 30 min per session. Participants in the ET and tsDCS + ET groups received the intervention under remote supervision by physiotherapists and kinesiologists, who configured and monitored individualized exercise programs according to each patient’s functional status and performance. The ET protocol consisted of a sequence of free-body exercises, including movements targeting the trunk (e.g., lateral bending and forward flexion) as well as the upper and lower limbs (e.g., shoulder abduction and knee flexion), preceded by a brief warm-up. Exercise intensity and volume were individually tailored, as the program was progressively adjusted by clinicians throughout the 3-week intervention period according to participants’ performance and functional capacity. The system consisted of a wearable inertial sensor and a tablet-based application that guided participants through the prescribed exercises. Movements were recorded in real time and transmitted via Bluetooth to the application, which provided real-time visual and auditory feedback on movement execution. Participants were trained individually in the use of the system prior to the start of the intervention to ensure independent and correct use. Adherence and performance were remotely monitored by clinical staff through a dedicated web-based platform, allowing quantitative tracking of treatment compliance and effectiveness.

### 2.5. Clinical Outcomes

Clinical outcomes were assessed using validated self-report scales covering multiple domains, including pain intensity, pain-related interference, health-related quality of life, anxiety and depression symptoms. These assessments were administered through the tablet-based application integrated into the digital rehabilitation system (Kari, Euleria, Trento, Italy), which was also used for the delivery of exercise sessions and the remote monitoring of treatment adherence and performance throughout the study.

Pain intensity was evaluated using the Visual Analogue Scale (VAS) [49], a 10 cm horizontal line on which participants indicate their perceived level of pain, ranging from “no pain” to “worst imaginable pain”.

Pain severity and its impact on daily functioning were assessed with the Brief Pain Inventory (BPI), a self-reported questionnaire, which provides measures of both pain severity (BPI-S) and pain interference in daily life (BPI-I). Each item is rated on an 11-point Likert scale ranging from 0, indicating no pain or no interference, to 10, indicating worst pain or maximum interference [50,51].

Health-related quality of life was measured using the Short-Form Health Survey (SF-12), a multidimensional instrument providing physical (PCS) and mental (MCS) component summary scores [52].

Anxiety and depressive symptoms were assessed using the Hospital Anxiety and Depression Scale (HADS), a 14-item self-report questionnaire with two subscales assessing anxiety (HADS-A) and depression (HADS-D), each ranging from 0 to 21 [53].

### 2.6. Statistical Analysis

Descriptive statistics were calculated for demographic and clinical characteristics, as well as for active medication intake at baseline. Between-group differences were assessed using Kruskal–Wallis tests for continuous variables and Fisher’s exact tests for categorical variables. Linear mixed-effects (LME) models were fitted to test the treatment-related effect across the four timepoints on each clinical outcome. LME models were followed by analysis of variance for *p*-values extraction. The models were specified using Wilkinson’s notation as follows:

*Y* ~ 1 + Group × Time + Age + Medication Intake + Disease Duration + (1|Subject) (1)

where *Y* was either HADS-A, HADS-D, SF-12-PCS, SF-12-MCS, BPI-S, BPI-I, or VAS score. Among the fixed-effect factors, *Group* indicated whether a participant received tsDCS, ET, or multimodal (tsDCS + ET) treatment, whereas *Time* reflected the timepoint when the clinical assessment was conducted (i.e., T0, T1, T2, or T3). An interaction term between these two factors was also included in the model. *Subject* was specified as a random effect factor. Participants’ *Age*, *Medication Intake* (i.e., whether they reported taking medication at baseline or not), and *Disease Duration* (in years) were included as covariates. Multicollinearity among predictors was assessed via variance inflation factor (VIF) examination. All VIF values were <2.00, indicating no evidence of problematic intercorrelations [54]. Residuals of the models and clinical outcomes across the four timepoints proved to be normally distributed, as indexed both by skewness and kurtosis reference values <|1| and <|3|, respectively [55], and by the absence of visual abnormalities in Q-Q plots and histograms. Post hoc comparisons were performed based on estimated marginal means (*EMMs*). Pairwise contrasts were computed between groups at each timepoint and between timepoints within each group, with *p*-values adjusted for multiple testing using the Bonferroni–Holm method [56]. Partial eta squared (η^2^) and Cohen’s *d* were employed as effect size measures. An a priori power analysis was also conducted using G*Power (Version 3.1) to determine the required sample size for detecting a significant within-between group interaction, confirming a total of 45 participants (i.e., 15 per group; effect size *f* = 0.25, α = 0.05, desired power (1 − β) = 0.95, 3 groups, 4 repeated measurements, *r* = 0.50 among repeated measures, nonsphericity correction ε = 1) and a critical *F*-value of 2.17 (with 6 and 126 degrees of freedom, respectively, and a noncentrality parameter of λ = 22.50). The cutoff for statistical significance was set at *p* < 0.05, 2-tailed. All data analyses were conducted using custom algorithms developed in R (Version 4.5.2; https://www.r-project.org/, accessed on 1 July 2026).

## 3. Results

Group-specific descriptive statistics were calculated for demographic variables, clinical characteristics, and current pharmacological treatment. The tsDCS, ET, and tsDCS + ET groups did not differ significantly in age (*χ*^2^(2) = 1.70, *p* = 0.428) or disease duration (*χ*^2^(2) = 1.47, *p* = 0.479). Moreover, no significant associations were found between group allocation and sex assigned at birth (*p* = 0.762) or ongoing pharmacological treatment (*p* = 0.770) (see Table 1).

**Figure 1:**
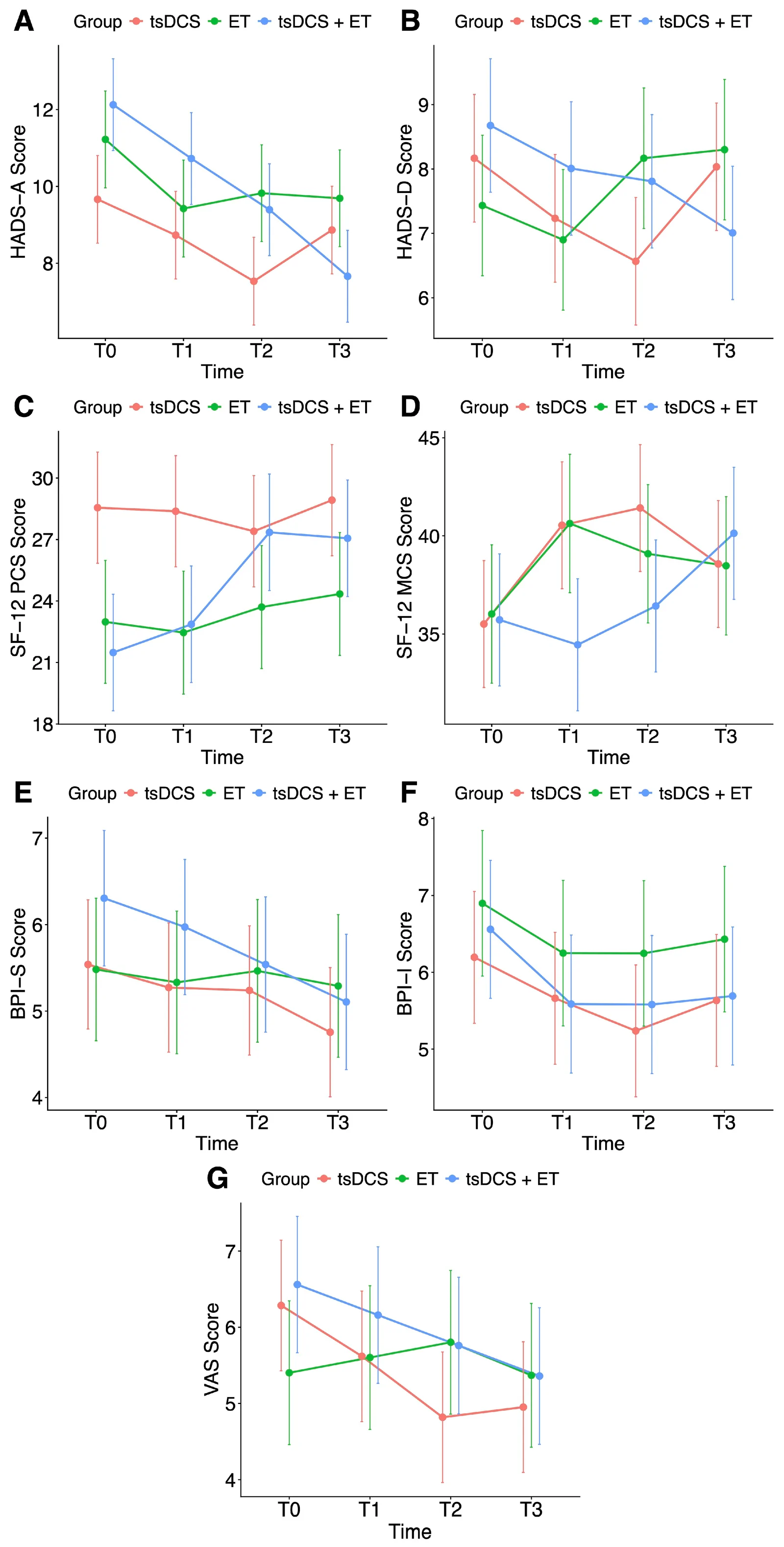
Treatment-related effects over time on clinical outcomes. Linear mixed-effect models were fitted to data to estimate the effect of tsDCS, ET, and multimodal treatment (tsDCS + ET) on each clinical outcome across the four timepoints. Line plots show the estimated marginal means and standard errors of each group over time. As unimodal treatment, tsDCS decreased anxiety at T2 compared to baseline, whereas tsDCS + ET decreased anxiety at both T2 and T3 compared to baseline (**A**). Global differential changes were detected in depression levels over time among the three treatment groups (**B**). The integration of tsDCS with ET increased subjects’ physical-related quality of life at both T2 and T3 compared to baseline (**C**), yet no differential effect of treatment on health-related quality of life was observed (**D**). TsDCS, ET and tsDCS + ET showed no differential effects over time on subjects’ perceived pain severity (**E**) and interference with their daily functioning (**F**). Regardless of treatment, a potentially significant decrease in VAS scores was detected over time (**G**).

A statistically significant group-by-time interaction effect was observed when the HADS-A score was set as a dependent variable (*F* (6, 126) = 3.37, *p* = 0.004, partial η^2^ = 0.138), indicating differential changes in anxiety-related symptom severity over time among the three treatment groups (see Figure 1A). Post hoc pairwise comparisons showed that tsDCS significantly decreased subjects’ anxiety at T2 (*EMM* = 6.74; *SE* = 0.91; *p* = 0.030, *d* = 1.04) compared to baseline (*EMM* = 8.88; *SE* = 0.91), while tsDCS + ET significantly decreased subjects’ anxiety at both T2 (*EMM* = 8.55; *SE* = 0.91; *p* = 0.002, *d* = 1.34) and T3 (*EMM* = 6.81; *SE* = 0.91; *p* < 0.001, *d* = 2.19) compared to baseline (*EMM* = 11.28; *SE* = 0.91). Likewise, a statistically significant group-by-time interaction effect was detected when the HADS-D score was used as a response variable (*F* (6, 126) = 2.46, *p* = 0.028, partial η^2^ = 0.105), suggesting overall differential changes in depression-related symptom severity over time among the three treatment groups (see Figure 1B). However, such an interaction effect could not be localized to specific between- or within-group contrasts after correction for multiple comparisons. A statistically significant main effect of *Time* was also observed for HADS-A (*F* (3, 126) = 2.79, *p* = 0.043, partial η^2^ = 0.062), whereas the main effect of *Time* on HADS-D did not reach statistical significance (*F* (3, 126) = 2.36, *p* = 0.075, partial η^2^ = 0.053).

Neither the group-by-time interaction effect for the SF-12-PCS score (*F* (6, 126) = 2.00, *p* = 0.070, partial η^2^ = 0.087) nor that for the SF-12-MCS score (*F* (6, 126) = 1.19, *p* = 0.318, partial η^2^ = 0.053) reached statistical significance (see Figure 1C,D). Nevertheless, post hoc pairwise comparisons showed that tsDCS + ET significantly increased subjects’ physical-related quality of life at T2 (*EMM* = 32.50; *SE* = 2.22; *p* = 0.004, *d* = 1.28) and T3 (*EMM* = 32.20; *SE* = 2.22; *p* = 0.006, *d* = 1.22) compared to baseline (*EMM* = 26.70; *SE* = 2.22).

Non-significant group-by-time interaction effects were detected for BPI-S (*F* (6, 126) = 0.48, *p* = 0.820, partial η^2^ = 0.023), BPI-I (*F* (6, 126) = 0.11, *p* = 0.995, partial η^2^ = 0.005), and VAS (*F* (6, 126) = 0.93, *p* = 0.476, partial η^2^ = 0.042) scores, indicating the absence of treatment-specific differential changes over time in subjects’ perceived pain severity and interference with their daily functioning (see Figure 1E–G). Furthermore, the main effect of *Time* on VAS scores did not reach statistical significance (*F* (3, 126) = 2.49, *p* = 0.063, partial η^2^ = 0.056), suggesting that, regardless of treatment, no statistically significant decrease in subjects’ perceived pain intensity occurred throughout the assessments. In no case did *Age*, *Medication Intake*, and *Disease Duration* show a significant association with the abovementioned clinical measures.

See Table 2 for a summary of the estimated scores for each clinical outcome across the three groups over time.

## 4. Discussion

This study aimed to investigate the effects of tsDCS, ET, and their combination on pain, psychological symptoms, and quality of life in individuals with FM. Overall, the findings suggest that while all interventions were associated with heterogenous degrees of improvement over time, the combined approach yielded more consistent and sustained effects on specific domains, particularly anxiety.

A significant group-by-time interaction was indeed observed for HADS-A scores, with the combined tsDCS + ET intervention leading to more sustained improvements compared to single treatments. While tsDCS alone was associated with a reduction in anxiety at the end of the intervention, this effect was not maintained at follow-up, whereas the combined treatment showed continued improvement over time. Such a pattern suggests that integrating neuromodulation with physical exercise may enhance both the magnitude and durability of treatment effects on affective symptoms. These findings are in line with previous evidence showing that non-invasive neuromodulation techniques combined with physical activity can modulate not only pain perception but also the affective dimension of chronic pain (see [45] for a review). In FM individuals, the combination of transcranial direct-current stimulation and aerobic exercise resulted in greater improvements in pain, anxiety, and mood compared with either intervention alone [57,58], suggesting additive effects of multimodal interventions targeting both neural and behavioural mechanisms involved in chronic pain [1]. At the same time, ET has been consistently associated with improvements in mood, stress regulation, and psychological well-being in chronic pain populations [31,59,60]. Hence, the present results extend this literature by suggesting that combining these approaches may mitigate the aberrant neuroplasticity mechanisms characterizing chronic pain [12] and, consequently, produce additive and synergistic effects, particularly in domains related to emotional functioning.

From a mechanistic perspective, the observed effects could reflect the complementary action of the two interventions. Neuromodulation techniques such as tsDCS are thought to influence central nociceptive processing through the modulation of spinal and supra-spinal excitability [15], as well as functional connectivity within pain-related networks [61]. At the molecular level, tsDCS can alter the balance between glutamatergic and GABAergic neurotransmission via intracellular signaling cascades [62]. In particular, spinal cord stimulation-induced changes in intracellular Ca^2+^ levels may activate the cAMP response element-binding protein [63,64,65], which in turn promotes the expression of activity-regulated cytoskeleton-associated protein [66]. Notably, the latter is involved in the activity-dependent trafficking and regulation of AMPA receptors [67], thereby contributing to alterations in glutamatergic transmission and long-term synaptic plasticity. In contrast, ET engages broader physiological systems, including endogenous analgesic pathways [36], autonomic regulation [68], and neuroendocrine responses [69]. The combination of these approaches may therefore act at multiple levels of the pain matrix, potentially resulting in more stable and generalized clinical benefits that extend beyond pain reduction to include improvements in the affective sphere.

In contrast, although a significant interaction effect was observed for depression, post hoc analyses did not reveal significant differences after correction for multiple comparisons. This evidence suggests that treatment-related effects on depressive symptoms may be weaker, more variable, or require longer intervention periods to become clinically meaningful for FM patients. Another explanation is that depressive symptoms in FM individuals are often associated with additional psychosocial factors that are not directly targeted by the interventions used in this study (e.g., pain catastrophizing, perceived stress, and maladaptive coping strategies) [70,71,72]. Also, the integration of physical activity with other non-invasive stimulation techniques, e.g., transcranial direct-current stimulation [73,74], could be employed to mitigate depressive symptoms in FM individuals while enabling a more focal modulation of brain networks involved in emotional processing.

Regarding quality of life, the results indicate a potential differential effect in the physical component, with significant improvements observed in the combined treatment group at both post-intervention and follow-up. This finding is consistent with previous studies highlighting the beneficial effects of neuromodulation and ET on physical functioning in FM individuals [58,75,76,77]. In contrast, no significant changes were observed in the mental component, indicating that broader aspects of mental well-being may require more targeted interventions (e.g., psychotherapy). Accordingly, guided imagery or cognitive behavioural therapy could represent valuable complementary approaches, as these interventions have demonstrated potential benefits in improving psychological symptoms and, in some cases, reducing pain-related outcomes in individuals with FM [78]. Another treatment strategy could be to combine ET (or neuromodulation) with pharmacotherapy, as adding home-based postural exercise to duloxetine has been shown to improve not only the physical component of quality of life but also sleep quality and trunk mobility in patients with FM [79].

No treatment-specific effects were detected for pain intensity or pain-related interference, although a general clinical improvement over time was observed across all groups. This finding may reflect the multifactorial nature of pain in FM patients [80,81], where symptom fluctuations and non-specific effects (e.g., increased clinical attention or expectation) likely contribute to perceived improvement. Alternatively, the duration or intensity of the interventions was not sufficient to produce detectable between-group differences in pain-related outcomes. Importantly, the dissociation between improvements in clinical outcomes and the absence of differential effects on pain-related measures supports the notion that pain intensity, functional impairment, and emotional symptoms represent partially independent domains that could respond differently to treatment. From a clinical perspective, this highlights the importance of adopting multimodal and multidimensional treatment approaches, as well as comprehensive outcome assessments that capture both physical and psychological aspects of the patient experience.

The present study, however, presents limitations. First, the absence of blinding and sham-controlled conditions may have introduced potential biases related to expectancy or placebo effects. Second, differences in intervention frequency across groups could have affected the comparability of treatment effects. Third, the reliance on self-reported measures, although widely validated, may be subject to reporting bias. An additional pivotal aspect concerns the home-based delivery of ET. On one hand, this approach enhanced treatment accessibility and ecological validity by enabling participants to engage in rehabilitation within their daily environment, while remote supervision through a digital platform ensured continuous monitoring of adherence and performance, thereby mitigating the limitations of unsupervised home-based interventions. On the other hand, the lack of direct in-person supervision may have introduced variability in exercise execution, which should be considered when interpreting the results. Fourth, although a power analysis was conducted to estimate the required number of participants, the sample size remains relatively small and the follow-up duration is limited to 3 months, suggesting that findings should be interpreted with caution in terms of durability and generalizability.

Overall, the study provides preliminary evidence supporting the potential added value of combining neuromodulation and ET in FM individuals. Future studies with larger samples, standardized intervention protocols, and longer follow-up periods (e.g., at 6, 12, and 24 months) are warranted to further investigate the potential synergistic effects of these interventions and to clarify their impact on different dimensions of the disorder.

## 5. Conclusions

The present study suggests that combining tsDCS with ET may provide added benefits to FM patients, particularly in reducing anxiety symptoms. Although no treatment-specific effects were observed for pain intensity and quality of life, our findings highlight the need of targeting both psychological and functional domains. These results support the potential role of multimodal interventions in the management of FM and warrant further investigation in larger and controlled studies.

## Figures and Tables

**Table 1:** Means and standard deviations of baseline demographic, clinical, and treatment-related characteristics of the tsDCS, ET, and tsDCS + ET groups.

Variable	tsDCS	ET	tsDCS + ET
N Age Sex (Male/Female)	15	15	15
	51.73 ± 13.99	52.27 ± 10.88	48.53 ± 12.03
	2/13	0/15	1/14
Disease Duration (Years)	9.73 ± 8.11	5.67 ± 3.94	7.07 ± 7.08
Pharmacological Treatment (Yes/No)	10/5	12/3	10/5
Psychotropic drugs Anxiolytics Antidepressants Antipsychotics			
	3	3	1
	7	6	4
	0	0	1
Hypnotics Central pain modulators	0	2	0
		
Opioids	3	3	0
Cannabinoids	1	1	1
Antiepileptics	6	7	4
Peripheral pain modulators			
Anti-Inflammatories	1	3	1
Pain Relivers	0	1	0
Analgesics	0	0	1
Others			
Myorelaxants	1	0	1
Anesthetics	0	0	1
Homeopathics	0	0	1

Abbreviations: tsDCS, transcutaneous spinal direct-current stimulation; ET, exercise therapy.

**Table 2:** Estimated marginal means and standard errors for each clinical outcome in the tsDCS, ET, and tsDCS + ET groups at baseline (T0), T1, T2, and T3.

Treatment	Variable	T0	T1	T2	T3
tsDCS	HADS				
	Anxiety	8.88 ± 0.91	7.94 ± 0.91	6.74 ± 0.91	8.08 ± 0.91
	Depression	8.29 ± 0.79	7.36 ± 0.79	6.69 ± 0.79	8.16 ± 0.79
	SF-12				
	PCS	33.00 ± 2.22	32.80 ± 2.22	31.80 ± 2.22	33.30 ± 2.22
	MCS	36.60 ± 2.65	41.60 ± 2.65	42.50 ± 2.65	39.70 ± 2.65
	BPI				
	Severity	5.38 ± 0.59	5.12 ± 0.59	5.08 ± 0.59	4.60 ± 0.59
	Interference	5.64 ± 0.69	5.11 ± 0.69	4.68 ± 0.69	5.08 ± 0.69
	VAS Score	6.17 ± 0.69	5.50 ± 0.69	4.70 ± 0. 69	4.84 ± 0.69
ET	HADS				
	Anxiety	10.44 ± 0.96	8.64 ± 0.96	9.04 ± 0.96	8.91 ± 0.96
	Depression	7.70 ± 0.83	7.16 ± 0.83	8.43 ± 0.83	8.56 ± 0.83
	SF-12				
	PCS	29.10 ± 2.35	28.50 ± 2.35	29.80 ± 2.35	30.40 ± 2.35
	MCS	36.80 ± 2.76	41.40 ± 2.76	39.80 ± 2.76	39.20 ± 2.76
	BPI				
	Severity	5.35 ± 0.62	5.20 ± 0.62	5.34 ± 0.62	5.16 ± 0.62
	Interference	6.28 ± 0.73	5.63 ± 0.73	5.63 ± 0.73	5.81 ± 0.73
	VAS Score	5.35 ± 0.72	5.55 ± 0.72	5.75 ± 0.72	5.31 ± 0.72
tsDCS + ET	HADS				
	Anxiety	11.28 ± 0.91	9.88 ± 0.91	8.55 ± 0.91	6.81 ± 0.91
	Depression	8.85 ± 0.79	8.18 ± 0.79	7.98 ± 0.79	7.18 ± 0.79
	SF-12				
	PCS	26.70 ± 2.22	28.00 ± 2.22	32.50 ± 2.22	32.20 ± 2.22
	MCS	36.80 ± 2.64	35.50 ± 2.64	37.50 ± 2.64	41.20 ± 2.64
	BPI				
	Severity	6.15 ± 0.59	5.81 ± 0.59	5.38 ± 0.59	4.95 ± 0.59
	Interference	5.95 ± 0.69	4.97 ± 0.69	4.97 ± 0.69	5.08 ± 0.69
	VAS Score	6.45 ± 0.69	6.05 ± 0.69	5.65 ± 0.69	5.25 ± 0.69

Abbreviations: tsDCS, Transcutaneous Spinal Direct-Current Stimulation; ET, Exercise Therapy; HADS, Hospital Anxiety and Depression Scale; SF-12, Short-Form Health Survey; PCS, Physical Component Summary; MCS, Mental Component Summary; BPI, Brief Pain Inventory; VAS, Visual Analogue Scale.

## Data Availability

The data presented in this study are available on request from the corresponding authors due to the multicentered nature of the study and related data agreements.

## Abbreviations

The following abbreviations are used in this manuscript:

AMPA	α-Amino-3-Hydroxy-5-Methyl-4-Isoxazolepropionic Acid
BPI	Brief Pain Inventory
BPI-I	Brief Pain Inventory–Pain Interference subscale
BPI-S	Brief Pain Inventory–Pain Severity subscale
cAMP	Cyclic Adenosine Monophosphate
EEG	Electroencephalography
EMMs	Estimated Marginal Means
ET	Exercise Therapy
FM	Fibromyalgia
GABA	Gamma-Aminobutyric Acid
HADS	Hospital Anxiety and Depression Scale
HADS-A	Hospital Anxiety and Depression Scale–Anxiety subscale
HADS-D	Hospital Anxiety and Depression Scale–Depression subscale
LME	Linear Mixed-Effects (models)
MCS	Mental Component Summary (score)
PCS	Physical Component Summary (score)
SE	Standard Error
SF-12	Short-Form Health Survey (12-item version)
tsDCS	Transcutaneous Spinal Direct-Current Stimulation
VAS	Visual Analogue Scale
VIF	Variance Inflation Factor
